# Remote Research Methods: Considerations for Work With Children

**DOI:** 10.3389/fpsyg.2021.703706

**Published:** 2021-10-28

**Authors:** Michelle M. Shields, Morgan N. McGinnis, Diana Selmeczy

**Affiliations:** Cognitive Development Lab, Department of Psychology, University of Colorado, Colorado Springs, Colorado Springs, CO, United States

**Keywords:** remote research, remote research design, remote research software, development, children

## Abstract

The growing shift to online research provides numerous potential opportunities, including greater sample diversity and more efficient data collection. While online methods and recruitment platforms have gained popularity in research with adults, there is relatively little guidance on best practices for how to conduct remote research with children. The current review discusses how to conduct remote behavioral research with children and adolescents using moderated (i.e., real-time interactions between the experimenter and child) and unmoderated (i.e., independent completion of study without experimenter interaction) methods. We examine considerations regarding sample diversity and provide recommendations on implementing remote research with children, including discussions about remote software, study design, and data quality. These recommendations can promote the use of remote research amongst developmental psychologists by contributing to our knowledge of effective online research practices and helping to build standardized guidelines when working with children.

## Introduction

Researchers have grown increasingly interested in collecting data using online or remote methodologies. Remote research provides several benefits, such as the potential for quicker data collection and the inclusion of more diverse participant samples (Buhrmester et al., 2011; Dworkin et al., 2016). However, remote methods may also present unique challenges, including difficulties transferring in-person studies to remote formats and the potential for lower quality data due to less direct control over the environmental setting (Bridges et al., 2020; Chmielewski and Kucker, 2020). Previous research using remote methods has mainly been conducted with adults (Paolacci and Chandler, 2014; Lee et al., 2018), and we have a limited understanding of how to best implement remote methodologies with developmental populations (Sheskin et al., 2020). Research conducted with children versus adults can vary substantially, such as differences in instructions and task design (Barker and Weller, 2003). Therefore, it is important to develop appropriate remote research practices that apply to developmental populations. Below we explore remote research methodologies with typically developing child and adolescent populations, focusing on behavioral research in cognitive psychology.

Before assessing the use of remote methods, it is important to note that remote research can be conducted in multiple formats. Unmoderated remote studies utilize online software that allows participants to complete a study individually, at any time, without the presence of a researcher. In contrast, moderated remote studies take place virtually such that researchers interact directly with participants through virtual meeting platforms (e.g., Zoom) and lead participants through the study procedure in real-time. We will include general considerations regarding both remote unmoderated and moderated methods to help investigators understand the benefits and drawbacks of each format.

## Benefits of Remote Research and Collecting Representative Samples

Diverse samples are critical to developmental research given the large amount of variability that occurs within developmental processes, including cognitive skills (Rowley and Camacho, 2015; Nielsen et al., 2017). Furthermore, developmental processes may be susceptible to environmental effects and vary as a function of ethnicity, socioeconomic status (SES), and geographical location (Bradley and Corwyn, 2002; McCulloch, 2006; Quintana et al., 2006). However, psychological research tends to collect data from homogenous or non-representative samples (Rowley and Camacho, 2015), and this may occur, in part, because most academic research uses in-person or lab-based studies. In-person studies may limit the diversity of research samples due to geographical, temporal, and fiscal restrictions (Rowley and Camacho, 2015; Nielsen et al., 2017; Rhodes et al., 2020). Importantly, remote methods have the potential to overcome some of these limitations by removing the time and costs associated with traveling to a physical research location and allowing individuals to participate at any time (in unmoderated studies). Consistent with this idea, some research shows that both adult and children samples collected through remote research have greater racial and geographical diversity than in-person studies (Birnbaum, 2004; Rhodes et al., 2020).

Despite the potential to increase sample diversity through remote research methods, diversity may still be limited for multiple reasons. For example, internet access, computer access, and technological literacy, which are frequently required to participate in remote studies, are often raised as critical barriers to participation (Kraut et al., 2004; Scott et al., 2017; Grootswagers, 2020). Furthermore, although remote research may decrease the need for travel, having children participate in remote studies may still be time-consuming for parents. For example, parents may need to answer scheduling emails, prescreening forms, or questionnaires, and provide consent or help during the study session. Thus, although remote studies have the potential to increase diversity, there are still limiting factors and future research is needed to determine whether the use of remote research can successfully increase diversity in child samples.

## Implementing Remote Research Studies

Research with children is generally considered more challenging than research with adults because tasks need to be adapted to appropriately match children’s language, comprehension, and executive function abilities, and children tend to be more subject to fatigue during participation (Fargas-Malet et al., 2010; Rollins and Riggins, 2021). Similar to in-person research, including engaging, meaningful, and easy to understand task content (Fargas-Malet et al., 2010; Nussenbaum et al., 2020) is also important when conducting remote research with children. Furthermore, although researchers can remotely collect physiological measures, including eye movements (Scott et al., 2017), most remote work collects behavioral responses. There are some instances when remote research may not be possible, such as when special equipment (e.g., EEG) or highly controlled environmental contexts are required. Below we outline considerations regarding software, experimental design, and data quality for remote behavioral research using typically developing children.

### Remote Technology

Remote behavioral research typically requires software that participants can interact with on their own devices (e.g., mobile phones, tablets, laptops, or computers). Several software and online platforms exist to aid researchers in remote data collection (see Table 1). A complete summary of available software is beyond the scope of the current review. We suggest researchers examine available software to select the one that best fits their needs. For example, some programs are available through an internet browser (e.g., Qualtrics, Gorilla Experiment Builder), while other programs may require participants to install software on specific operating systems or devices (e.g., Eprime Go). Online software may also vary in its flexibility to implement research designs. For example, Qualtrics is commonly used to collect survey responses but has limited functions for complex coding (e.g., randomization based on multiple variables).

**TABLE 1:** Comparison of remote software.

	**Gorilla**	**Inquisit Web**	**PsyToolkit**	**EPrime3/Eprime-Go**	**Qualtrics**	**Psychstudio**	**PsychoPy3/PsychoJs**
Remote platform	Web-based	Web-based* -Includes offline feature through application *Requires local download by researcher for customization	Web-based	Web-based* *Requires local download by researcher to create study, may require local download by participant for running study, supported only by Windows OS	Web-based -Includes offline feature through application	Web-based	Web-based* *Requires local download by researcher for creating study and additional software (Pavlovia) to run online
Programming language	Typescript (super-set of Javascript) and Handlebars (HTML templating engine)	Similarities to HTML/XML and C-family of languages	Custom scripting language, C- family languages	Custom object- oriented scripting language (E-Basic)	HTML, CSS, and JavaScript	No custom code available	Python
Input measures	Mouse Keyboard Audio recording Video recording Mouse- tracking Eye-tracking	Mouse Keyboard Audio recording	Mouse Keyboard	Mouse Keyboard	Mouse Keyboard	Mouse Keyboard	Mouse Keyboard
Pricing model	Free to use Pay per participant	License fee	Free	License fee	Free basic version License fee for full version	License Fee	Free
Additional supported devices	Mobile Tablet	Mobile Tablet	Mobile Tablet	Tablet	Mobile Tablet	Mobile Tablet	Mobile Tablet

*This table is not an exhaustive list and additional features may be available.*

Researchers who work with children and adolescents should also consider participants’ development capabilities regarding technology use when designing remote studies. Although more research is needed on children’s evolving technological skills, direct observations of children’s interaction with technology show that toddlers (Geist, 2012) and infants as young as 15-months-old are able to tap on touch screen devices (Zack et al., 2009; Ziemer et al., 2021). Preschoolers can engage in more complex touch actions such as drag-and-drop (Vatavu et al., 2015). Furthermore, both direct observations and parental reports suggest that 2.5-year-olds begin to use a mouse or keyboard input and 5-year-olds begin to develop basic typing skills with substantial improvements throughout middle childhood (Read et al., 2001; Calvert et al., 2005; Donker and Reitsma, 2007; Kiefer et al., 2015). Therefore, researchers must adopt technological methods that can accommodate the fine-motor skills of their participants, such as using mobile devices or tablets to collect touch input when working with younger children. Researchers should also consider using software that enables video/audio recordings (e.g., Gorilla) or using video conference programs (e.g., Zoom) to collect verbal rather than typed responses for younger participants. Furthermore, children’s previous experience with technological devices can also impact research findings (Couse and Chen, 2010; Jin and Lin, 2021), suggesting that researchers should measure children’s familiarity with technology as a potential covariate.

Differences in hardware, software, and response modality may also impact the precision and accuracy of display times, location of stimuli, or response times (Chetverikov and Upravitelev, 2016; Poth et al., 2018). Although remote research software has relatively minimal display and reaction time delays (<100 ms) (Anwyl-Irvine et al., 2020; Bridges et al., 2020), variability exists between browsers, operating systems, and hardware (Garaizar and Reips, 2019; Bridges et al., 2020). Additionally, certain input types (e.g., touch) can result in faster reaction times compared to other input modalities (e.g., mouse) (Woodward et al., 2017; Ross-Sheehy et al., 2021), suggesting it is important to control for input type when measuring reaction times. Critically, general findings may replicate across study methods, with recent research suggesting that response time patterns in children ages 4–12 are similar across remote and in-person studies (Nussenbaum et al., 2020; Ross-Sheehy et al., 2021). Overall, researchers who need highly precise stimuli presentation or response times should instruct participants to use a particular setup during study sessions (e.g., Chrome browser and keyboard), calibrate programs to adjust for the type of operating system and device used, and use within-subjects comparisons or controls (Bridges et al., 2020).

### Study Design and Data Quality

Researchers have less control over the experimental environment during remote research, potentially lowering data quality. Remote methods can differ from in-person research in terms of participant engagement (Dandurand et al., 2008), response honesty (Shapiro et al., 2013), and susceptibility to scammers (Dennis et al., 2018). We discuss these factors below and include recommendations on how to overcome some of these challenges.

#### Task Considerations and Instructions

Remote studies may result in fewer participant—researcher interactions, especially in unmoderated remote research. Although this may be less of a concern in research with adults, the cognitive skills required to independently guide oneself through a task, including self-regulation and language abilities, develop substantially throughout childhood (Montroy et al., 2016; Skibbe et al., 2019). Additionally, infants through preschoolers learn better from in-person interactions than pre-recorded videos (DeLoache et al., 2010; Myers et al., 2017). However, social exchanges that occur virtually in real time (e.g., video chatting) have been shown to be effective even for young children’s learning (Strouse and Samson, 2021). Therefore, moderated remote methods where virtual participant-research interactions occur may be especially appropriate with younger children. However, unmoderated methods are still possible when additional considerations are used, such as comprehensive instructions, comprehension checks, and parental involvement (Oppenheimer et al., 2009; Kannass et al., 2010; Scott et al., 2017). Furthermore, developmental differences in reading ability can be lessened by using age-appropriate, prerecorded instructions.

Parental involvement may increase during remote relative to in-person studies. For example, parents need to be able to operate and troubleshoot the technological software used for remote research. Because of this, we suggest the use of browser-based platforms and to limit the use of special software that requires local downloads (see Table 1). Furthermore, we suggest that prior to the study session, researchers provide parents with step-by-step instructions on how to use software (see https://osf.io/wahky/ for guides on using Zoom from our lab) and information on what type of hardware can be used (e.g., mobile phones, tablets, laptops). Critically, due to COVID-19, adults’ technological literacy (Sari, 2021) and children’s time spent interacting with technology has increased (Drouin et al., 2020; Ribner et al., 2021). These changes have likely made it easier for parents and children to implement basic functions in video conferencing platforms (e.g., video/audio communication and screen sharing) and other software. However, we recommend that researchers add approximately 10 min of additional time during study sessions to troubleshoot any technological issues and prepare to reschedule sessions if needed.

Researchers may also want to intentionally direct parental involvement during data collection. Parental support and scaffolding can be helpful, especially when working with younger children. Recent research shows that during remote sessions having parents input responses for children ages 4–10 results in similar findings as in-person studies (Ross-Sheehy et al., 2021), providing some evidence that parental involvement can be used successfully during remote research. However, researchers may often want to prevent unwanted parental involvement (e.g., additional unmonitored explanations, biasing of responses) or require children to input their own responses, especially if accurate response times are needed. As children learn to communicate independently, they may be less likely to need parental intervention, with research suggesting children as young as 4 years of age can independently input their responses during remote research (Vales et al., 2021). To limit parental involvement during data collection, researchers can read instructions to children or use pre-recorded audio or videos (Rhodes et al., 2020). During moderated sessions, researchers could also share their screen and input children’s responses or have children share their screen and monitor children’s behaviors while children input their own responses. We also recommend that researchers communicate to parents the importance of children’s independent responses. Additionally, we suggest researchers collect feedback from both children and parents on any issues that may have come up during the study, such as cheating or asking for parental help.

#### Increasing Attention and Motivation

Lack of participant attention during remote research, including increased distractions and decreased motivation, can lower data quality (Zwarun and Hall, 2014; Finley and Penningroth, 2015). Participants are also more likely to experience distractions in natural settings outside of a research laboratory, and these distractions can lead to different findings than those observed during lab-based studies (Kane et al., 2007; Varao-Sousa et al., 2018). Furthermore, children and adolescents have greater difficulty ignoring irrelevant information (Davidson et al., 2006; Garon et al., 2008), and therefore environmental distractions may be more likely to impact remote research with developmental populations. In addition to distractions, it is possible that participants may be less motivated during remote studies and rapidly complete tasks or provided unvaried answers (Litman et al., 2015; Ahler et al., 2020).

Several methods have been found to reduce participant inattention during remote research with adults. Attention checks, including trap questions (e.g., regardless of your true preference select “Movies”) can be used to flag inattentive participants (Liu et al., 2009; Hunt and Scheetz, 2018). Comprehension checks (e.g., what are your instructions for this task?) can also be used to help researchers ensure that participants understand and are attentive to the task. Researchers can then use predetermined criteria for removing participants based on responses to these questions to improve overall data quality (Dworkin et al., 2016; Jensen-Doss et al., 2021). When working with children, trap questions (e.g., answer this question by pressing the blue button) and comprehension checks (e.g., select the option that shows what you will be doing in the study) that require specific age-appropriate responses can also be included to assess and remove inattentive participants. Moderated studies with children may be inherently more engaging and therefore less susceptible to low levels of attention and motivation (Dandurand et al., 2008), but researchers can still continue to directly monitor, address, and note participant attention. Additionally, shorter, engaging tasks may improve attention during remote research, including the use of animations, child-friendly stimuli, and frequent breaks (Barker and Weller, 2003; Rhodes et al., 2020).

#### Limiting Cheating

Another concern that can affect data quality is the honesty of participants’ responses. Participants may be more likely to answer dishonestly on tasks completed in the absence of researcher supervision (Lilleholt et al., 2020). The percentage of adults that cheat during online studies can vary (e.g., ranging from 24 to 41% according to Clifford and Jerit, 2014), but research suggests that most adult participants answer honestly when encouraged to do so (Corrigan-Gibbs et al., 2015). However, cheating behaviors may differ when working with developmental populations, with research suggesting younger children ages 8–10 cheat more frequently than older children ages 11–16 during in-person studies (Evans and Lee, 2011; Ding et al., 2014).

Several methods have been shown to decrease cheating behaviors. Simple interventions such as honesty reminders (e.g., “please answer honestly”) and honesty checks were found to decrease cheating behaviors in adults (Clifford and Jerit, 2014; Corrigan-Gibbs et al., 2015) and children (Heyman et al., 2015), and these types of interventions can easily be included in either moderated or unmoderated remote research. During unmoderated sessions, researchers could also minimize cheating by recording participants or taking periodic video captures of participants. During moderated sessions, researchers can monitor participants via video and screen-sharing, and verbally intervene if cheating behaviors are observed. In our own remote research, we have found that nearly all families consent to video recording (>99%) during moderated sessions, suggesting video monitoring is a potentially feasible solution to help mitigate cheating (see https://osf.io/hrp4y/ for our consent documents). Finally, task designs may need to be altered to mitigate cheating, particularly during memory tasks during which cheating can easily occur (e.g., writing down to-be-learned material). To minimize cheating, memory researchers can avoid stimuli that can easily be labeled and instead use abstract, similar, or difficult to label stimuli (e.g., scenes, fractals), limit encoding time and require participants to complete an additional task during encoding (e.g., mouse-click on the presented stimuli), or use incidental encoding designs where participants are unaware that an upcoming memory test will occur.

#### Avoiding Bots and Scammers

Remote studies with minimal researcher interaction may be at risk for compromised data quality due to information-security threats (Teitcher et al., 2015). Previous research with adults has highlighted information-security issues and offers potential solutions (Ahler et al., 2020; Chmielewski and Kucker, 2020). For example, automated computer program responses (i.e., bots) tend to differ from human responses and consist of atypical text formats, grammatically incorrect text responses, or responses that directly copy prompt text (Chmielewski and Kucker, 2020). Therefore, bots are relatively easy to flag and remove. Implementing bot checks (e.g., captchas) and collecting participant screening questions can also decrease bots (Jones et al., 2015; Kennedy et al., 2020). However, scammers may be particularly problematic for remote developmental research, especially during unmoderated designs. Scammers often fabricate responses to receive compensation (Chandler and Paolacci, 2017), including falsely claiming to be of a key demographic (e.g., an adult claiming to be a child). To alleviate some of these issues, researchers can utilize prescreening questions and check for consistencies in responses, such as asking about a child’s age repeatedly and in multiple formats (e.g., DOB, numeric age) or requesting specific information relevant to identifying your targeted population (e.g., asking a parent to describe a recent moment they were proud of their child) (Jones et al., 2015). Email requests to participate in a research study can also be monitored for potential scammers. Instances of strange email addresses, rapidly incoming email inquiries, and inquiries consisting of unusual responses (grammatical errors, copied text, etc.), may further indicate potential scammers. Ultimately, moderated studies may be the most effective at reducing scammers as direct participant-researcher interactions can easily ensure human participants are completing the study.

## Conclusion

As remote research becomes more common, understanding its benefits and limitations is increasingly important. Above, we outlined several considerations for implementing remote research with children and adolescents, including information about participant samples, remote technologies, study design, and data quality. Although future work is needed to better understand how remote research differs between children and adults, and which methods are most effective for children, the provided recommendations contribute to building a guideline for effective and reliable remote research with developmental populations.

## Author Contributions

MS contributed to the development and writing of the manuscript. MM contributed to the literature review process and editing of the manuscript. DS contributed to the development and revision of the manuscript. All authors read and approved the submitted version of the manuscript.

## Conflict of Interest

The authors declare that the research was conducted in the absence of any commercial or financial relationships that could be construed as a potential conflict of interest.

## Publisher’s Note

All claims expressed in this article are solely those of the authors and do not necessarily represent those of their affiliated organizations, or those of the publisher, the editors and the reviewers. Any product that may be evaluated in this article, or claim that may be made by its manufacturer, is not guaranteed or endorsed by the publisher.
